# Is Chytridiomycosis Driving Darwin’s Frogs to Extinction?

**DOI:** 10.1371/journal.pone.0079862

**Published:** 2013-11-20

**Authors:** Claudio Soto-Azat, Andrés Valenzuela-Sánchez, Barry T. Clarke, Klaus Busse, Juan Carlos Ortiz, Carlos Barrientos, Andrew A. Cunningham

**Affiliations:** 1 Laboratorio de Salud de Ecosistemas, Facultad de Ecología y Recursos Naturales, Universidad Andres Bello, Santiago, Chile; 2 Institute of Zoology, Zoological Society of London, Regent’s Park, London, United Kingdom; 3 Natural History Museum, Department of Life Sciences, Cromwell Rd., London, United Kingdom; 4 Zoologisches Forschungsmuseum Alexander Koenig, Bonn, Germany; 5 Departamento de Zoología, Facultad de Ciencias Naturales y Oceanográficas, Universidad de Concepción, Concepción, Chile; Clemson University, United States of America

## Abstract

Darwin’s frogs (*Rhinoderma darwinii* and *R. rufum*) are two species of mouth brooding frogs from Chile and Argentina that have experienced marked population declines. *Rhinoderma rufum* has not been found in the wild since 1980. We investigated historical and current evidence of *Batrachochytrium dendrobatidis* (*Bd*) infection in *Rhinoderma* spp. to determine whether chytridiomycosis is implicated in the population declines of these species. Archived and live specimens of *Rhinoderma* spp., sympatric amphibians and amphibians at sites where *Rhinoderma* sp. had recently gone extinct were examined for *Bd* infection using quantitative real-time PCR. Six (0.9%) of 662 archived anurans tested positive for *Bd* (4/289 *R. darwinii*; 1/266 *R. rufum* and 1/107 other anurans), all of which had been collected between 1970 and 1978. An overall *Bd*-infection prevalence of 12.5% was obtained from 797 swabs taken from 369 extant individuals of *R. darwinii* and 428 individuals representing 18 other species of anurans found at sites with current and recent presence of the two *Rhinoderma* species. In extant *R. darwinii*, *Bd*-infection prevalence (1.9%) was significantly lower than that found in other anurans (7.3%). The prevalence of infection (30%) in other amphibian species was significantly higher in sites where either *Rhinoderma* spp. had become extinct or was experiencing severe population declines than in sites where there had been no apparent decline (3.0%; *x* ^2^ = 106.407, *P*<0.001). This is the first report of widespread *Bd* presence in Chile and our results are consistent with *Rhinoderma* spp. declines being due to *Bd* infection, although additional field and laboratory investigations are required to investigate this further.

## Introduction

There are two species of Darwin’s frogs, both of which inhabit the temperate forests of South America: the northern Darwin’s frog (*Rhinoderma rufum*), which is endemic to central Chile, and the southern Darwin’s frog (*R. darwinii*), which is found in south and southern Chile and also in adjacent areas of Argentina [1], [2]. The behaviour that sets these frogs apart from all other amphibians is that the males care for their young by incubating them in their vocal sacs for at least part of their development, a process known as neomelia [3], [4]. In recent decades, both species have undergone marked population declines and *R. rufum* has not been recorded since 1980 [5]. The reasons for these apparent disappearances remain poorly understood [6], [7]. Throughout the historical distribution of *R. rufum*, and within the northern range of *R. darwinii*, there has been extensive habitat degradation, due to the large-scale replacement of native forest with pine (*Pinus radiata*) and eucalyptus (*Eucalyptus globulus*) plantations, and land use change to agriculture [1], [2]. Habitat loss, however, does not fully explain the enigmatic disappearances of *R. rufum* from its entire historical range or of the declines of *R. darwinii* from undisturbed ecosystems, including National Parks [8]. In this context, it has been hypothesised that amphibian chytridiomycosis, an infectious disease caused by the nonhyphal zoosporic chytrid fungus, *Batrachochytrium dendrobatidis* (*Bd*), might be implicated in the disappearances of Darwin’s frogs [1], [2], [8].

Amphibian chytridiomycosis, a recently-described emerging disease of amphibians [9], [10], has been associated with amphibian epizootic mass mortalities, population declines and global extinctions in different regions of the world [11], [12], [13], [14], [15], [16], [17]. Different genotypes of the fungus have been described, with the most virulent being a recombinant lineage, termed the global panzootic lineage (*Bd*GPL) [18]. Recently, *Bd* whole-genome sequencing has demonstrated a higher genetic differentiation than previously recognised (including within *Bd*GPL) [18], [19] and a complex evolutionary history that predates contemporary amphibian declines [20]. This highly-pathogenic and readily-transmissible pathogen appears to be capable of infecting an entire class of organism (the Amphibia), with devastating effects [21]. It has been described as: “the worst infectious disease ever recorded among vertebrates in terms of the number of species impacted and its propensity to drive them to extinction” [22]. In 2007, chytridiomycosis was identified as the cause of death of a group of 30 wild-caught *R. darwinii* exported to Germany for captive breeding [23]. Infection with *Bd* has been reported in populations of the invasive African clawed frog, *Xenopus laevis* [24] in central Chile. Additionally, Bourke et al. [25], [26] recently described *Bd* infection in *R. darwinii* and two other native frog species in the south of the country. The impacts of this emerging disease on amphibian populations in Chile, including Darwin’s frogs, however, have not been studied.

Here, we investigate whether amphibian chytridiomycosis is implicated in the population declines of Darwin’s frogs. We looked for evidence of historical *Bd* infection in *Rhinoderma* spp. and amphibians at current and former *Rhinoderma* sp. sites prior to and post the onset of declines. Also, we determined how widespread *Bd* infection is both in contemporary populations of *R. darwinii* across its current range and in other anuran species at sites of *Rhinoderma* spp. population decline or recent extinction.

## Materials and Methods

### Ethics statement

This study was carried out in strict accordance with the recommendations in the guidelines for use of live amphibians and reptiles in field research compiled by the American Society of Ichthyologists and Herpetologists (ASIH). Research was approved by the ZSL Ethics Committee and was conducted following Chilean and Argentinian wildlife regulations and according to permits 1241/08, 7377/09, 7993/10 and 300/12 of the Livestock and Agriculture Service (SAG) and 20/09, XI-01/09, 28/11 and X-03/11 of the National Forestry Corporation (CONAF) both in Chile, and permit 1119/11 of the National Parks Administration (APN) in Argentina. Archived amphibians were examined in their museum of origin, by the authors with specific permission given by all 5 zoological institutions.

### Study area

**Figure 1:**
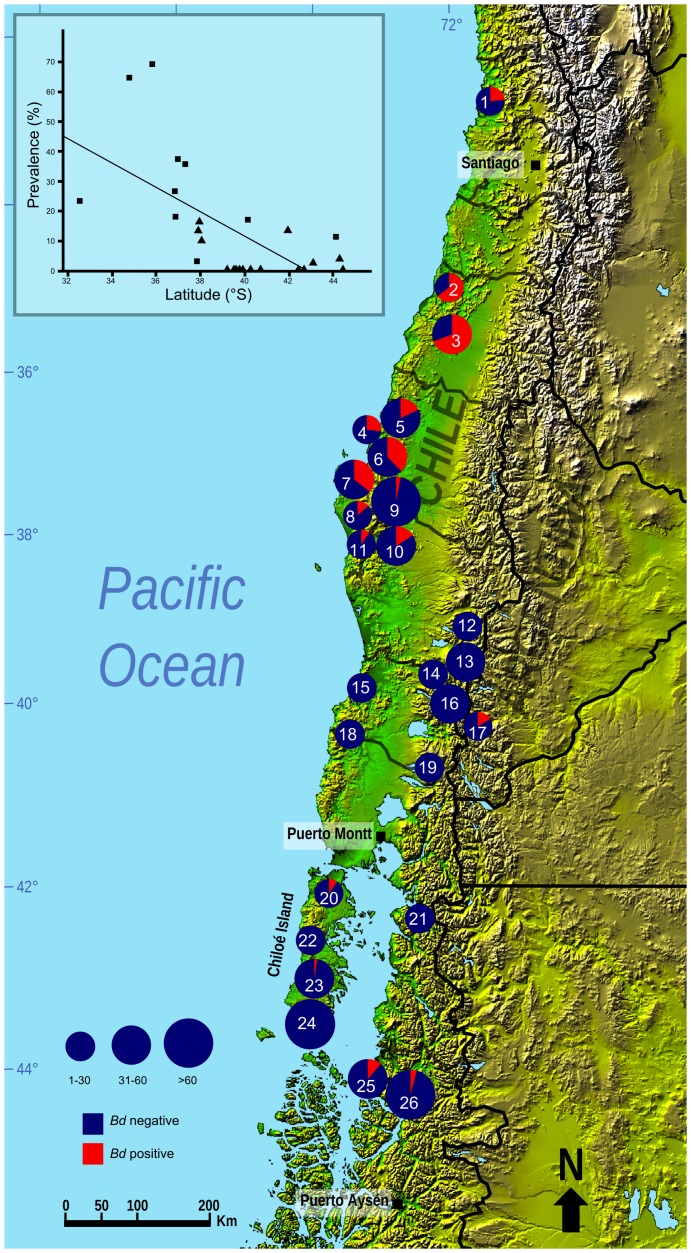
*Batrachochytrium dendrobatidis* infection prevalence at sites with extant or recently extinct *Rhinoderma* spp. Map of central-south Chile and Argentina showing sites from which *Rhinoderma* spp. and sympatric anurans were sampled for *Batrachochytrium dendrobatidis* (*Bd*) detection between 2008 and 2012. Sample size is represented by the size of the circles, with *Bd* prevalence shown in the red segments. Inset: Graph showing the relationship between latitude and prevalence of *Bd* infection by site (*R* ^2^ = 0.405, P<0.001). Squares: sites with recent extinction or population decline of *Rhinoderma* spp. Triangles: sites with extant populations and no evidence of population decline of *R. darwinii*.

Archived amphibian specimens from museum collections in Europe and Chile were examined for evidence of *Bd* infection. Also, extensive surveys for *Bd* infection throughout the historical ranges of *R. rufum* and *R. darwinii* were conducted from October 2008 to March 2012. These ranges extended from Zapallar (32° 33’ 03’’S, 71° 26’ 37’’W) to Aysén (45° 24’ 24’’S, 72° 41’ 52’’W) in Chile, and included adjacent areas in the Andes in the Neuquén and Río Negro Provinces in Argentina (Figure 1).

### Archived anurans

A retrospective study was carried out by examining 555 postmetamorphic *Rhinoderma* spp. and 107 sympatric anuran specimens, from the collections of the Zoologisches Museum Hamburg (ZMH, n = 321); Natural History Museum, London (BMNH, n = 142); Museo de Zoología, Universidad de Concepción, Chile (MZUC, n = 121); Zoologisches Forschungsmuseum Alexander Koenig, Bonn (ZFMK, n = 46); and Centro de Investigaciones Zoológicas, Universidad de Chile (CIZ, n = 32). Specimens were preserved in 70% ethanol (or 70% industrial methylated spirits for BMNH amphibians) and had been collected in central and south Chile between 1835 and 1989 (Table 1) for purposes other than disease investigation.

**Table 1:** Archived Darwin’s frogs (*Rhinoderma* spp.) and sympatric amphibians from European and Chilean museums examined for *Batrachochytrium dendrobatidis* (*Bd*) infection.

Species	Period of collection	No. examined	No. positives	*Bd* prevalence
*Batrachyla leptopus*	1869−1932	6	0	0
*Batrachyla taeniata*	1845−1972	38	0	0
*Calyptocephalella gayi*	1871−1978	21	0	0
*Nannophryne variegata*	1845−1962	17	0	0
*Pleurodema bufonina*	1971	2	0	0
*Pleurodema thaul*	1970−1972	9	1	11.1
*Rhinella spinulosa*	1844−1860	13	0	0
*Rhinella arunco*	1972	1	0	0
*Rhinoderma darwinii*	1835−1989	289	4	1.4
*Rhinoderma rufum*	1904−1980	266	1	0.4

### Living anurans

Cross-sectional studies were carried out at sites where *R. darwinii* was extant and at sites where *Rhinoderma* spp. had recently (since 1966) become extinct. Sites were delimited and a search effort of one hour by two researchers was conducted during daylight hours using a standardised methodology, as previously described [8].

### Sampling

**Archived anurans.** The skin of the ventral pelvis and ventral hind limbs of each amphibian museum specimen was sampled by brushing with a tapered inter-dental brush (3.2 to 6.0 mm; Oral B Laboratories), following Soto-Azat et al. [27]. Where multiple specimens were held in a single jar, they were rinsed with running tap water prior to sampling to remove possible surface contamination with *Bd*. Each specimen was handled using a new pair of disposable nitrile or latex gloves.

**Live anurans.** Only post-metamorphic and adult anurans were sampled. Frogs were captured by hand, safely contained in individual sealed plastic bags and put back immediately after the capture session in the exact place of capture. Each individual was handled with the use of clean disposable nitrile gloves. A sterile dry, rayon-tipped swab (MW100, Medical & Wire Equipment Co.) was firmly run five times each over the ventral abdomen and pelvis, each ventral hind limb (femur and tibia) and the plantar surface of each hind foot, to complete a total of 35 strokes. Dorsal and ventral pattern photographs were taken of each Darwin’s frog sampled for identification purposes. In order to minimize any *Bd* contamination of samples or the spread of pathogens within or between study sites by researchers, equipment or materials, a strict field sampling and disinfection protocol was followed according to that recommended by the Amphibian and Reptile Groups, UK: ARG Advice Note 4 (http://www.arguk.org/advice-and-guidance/view-category). All samples were stored at −80 °C until processed.

### Diagnostic analysis

Post sampling, whole interdental brushes and swab tips were deposited separately in 1.5 ml Eppendorf tubes containing 50 and 60 µl, respectively, of PrepMan Ultra (Applied Biosystems) and between 30 to 40 mg of Zirconium/silica beads of 0.5 mm diameter (Biospec Products). For each sample, DNA was extracted following the protocol of Boyle et al. [28]. Extracted DNA was diluted (1:10) in double-distilled water and analysed using a quantitative real-time polymerase chain reaction Taqman assay (qPCR) with primers specific for the ITS-1/5.8S ribosomal DNA region of *Bd*. In addition, bovine serum albumin (BSA) was included in the Taqman mastermix to minimise inhibition of the PCR [29]. For each sample, diagnostic assays were performed in duplicate, and standards of known zoospore concentration were included within each PCR plate, as were negative controls. A result was considered positive when: (1) amplification (i.e. a clearly sigmoid curve) occurred in both replicated PCR assays, (2) values higher than 0.1 genomic equivalents (GE) were obtained from both replicated reactions, and (3) average GE from both replicates were higher than its standard deviation. Extracted DNA from any positive sample was re-tested in duplicate and only determined to be positive for the purposes of this study if *Bd* DNA was clearly amplified in duplicate wells for a second time.

### Data analysis

Areas with historical and current presence of *Rhinoderma* spp. > 2 km from each other were determined to be separate sites or populations [30]. Statistical analyses were performed using SPSS (v. 20.0) to detect any significant difference between: 1) *Bd* prevalence and time in archived *R. darwinii* (using Fisher’s exact test for small sample sizes), 2) *Bd* prevalence in extant *R. darwinii* and sympatric amphibians (using the chi-squared test), 3) *Bd* intensity in extant *R. darwinii* and all other amphibian species tested (using the Mann-Whitney *U*-test), and 4) *Bd* prevalence at sites with and without evidence of recent *Rhinoderma* spp. population decline in extant *R. darwinii* (using the chi-squared test). Data on *Rhinoderma* spp. abundance is scarce. To consider a population having evidence of recent decline, we used data from a previous study [8], which investigated population sizes and the extent of declines in Darwin’s frogs. Briefly, populations categorised as having declined comprised those known to have disappeared since 1966, or (in one case) known to have undergone a recent marked population decline. A relationship between *Bd* prevalence at sites with historical and current *Rhinoderma* spp. populations and latitude was also tested using a simple linear regression model.

## Results

### Batrachochytrium dendrobatidis in archived amphibians

Six (0.9%) of 662 archived anurans were positive for *Bd* (4/289 *R. darwinii*; 1/266 *R. rufum*; and 1/107 sympatric anurans, a four-eyed toad, *Pleurodema thaul*). Each *Bd*-positive sample was positive in duplicate when re-tested. *Bd*-infected specimens were not equally distributed across time of collection, with all *Bd*-positive animals having been collected in or since 1970 (1835−1969 = 0/347, 1970−1989 = 6/315; Fisher’s exact test, *P* = 0.011). Details of species sampled, periods of collection and *Bd* positive individuals are presented in Tables 1 and 2.

**Table 2:** Historical presence of *Batrachochytrium dendrobatidis* (*Bd*) infection in *Rhinoderma* spp. and sympatric amphibians.

Year of Collection	Specimen reference no.^a^	Species	Origin	GE	SD
1970	BMNH 19.722.013	*P. thaul*	Concepción	0.2	0.0
1971	MZUC A36870	*R. darwinii*	PN V. Perez Rosales	0.1	0.1
1975	ZMH A04604	*R. rufum*	Chiguayante	0.4	0.1
1978	ZFMK 32088	*R. darwinii*	Valdivia	0.5	0.1
1978	ZFMK 32089	*R. darwinii*	Valdivia	0.6	0.0
1978	ZFMK 32091	*R. darwinii*	Valdivia	0.5	0.0

Details of *Bd* positive archived amphibians with number of genomic equivalents (GE) detected using a *Bd*-specific quantitative real-time PCR Taqman assay.

a BMNH  =  Natural History Museum, London; MZUC  =  Museo de Zoología, Universidad de Concepción, Chile; ZMH  =  Zoologisches Museum Hamburg; ZFMK  =  Zoologisches Forschungsmuseum Alexander Koenig, Bonn.

### Batrachochytrium dendrobatidis in extant amphibians

Twenty-six sites with current or past presence of *Rhinoderma* spp. were surveyed. No *R. rufum* was found. A total of 797 skin swabs were obtained from *R. darwinii* (n = 369) and other amphibians (428), including areas with current presence of *R. darwinii* (16 sites, 144 sympatric amphibians) and 284 amphibians from sites where *Rhinoderma* spp. had gone extinct since 1966 (10 sites). Details of *Bd* positive individuals by site were: (1) Zapallar, 4/17 (No. amphibian positive/No. tested); (2) Lago Vichuquén, 11/17; (3) Río Longaví, 25/36; (4) Chiguayante, 4/15; (5) San Pedro, 8/44; (6) Hualqui, 12/32; (7) Ramadillas, 14/39; (8) Butamalal, 2/15; (9) PN Nahuelbuta, 2/63; (10) El Natre, 5/31; (11) Contulmo, 2/20; (12) PN Huerquehue, 0/11; (13) PN Villarrica, 0/38; (14) Coñaripe, 0/14; (15) Oncol, 0/8; (16) Huilo Huilo, 0/37; (17) PN Lanín, 1/6; (18) PN Alerce Costero, 0/7; (19) PN Puyehue, 0/22; (20) Senda Darwin, 2/15; (21) Huinay, 0/15; (22) Los Alerzales, 0/13; (23) Tantauco Norte, 1/40; (24) Tantauco Sur, 0/130; (25) Melimoyu, 4/35; and (26) PN Queulat, 3/77 (Figure 1). We found *Bd* to be widespread in central-south Chile, from the region of Valparaiso to the region of Aysén, and also to be present in Argentina, covering an area of 1,305 km in length, with an estimated overall infection prevalence of 12.5%, varying by site from 0 to 69.4%. The prevalence of *Bd* infection varied amongst species, from 0 to 100% of individuals tested, although sample sizes for many species were small and distributed across multiple sites (Table 3).

**Table 3:** *Batrachochytrium dendrobatidis* (*Bd*) infection in 19 amphibian species at 26 sites with historical and current presence of Darwin’s frogs (*Rhinoderma* spp.), sampled during the period 2008−2012 in central and south Chile and south-western Argentina.

Species	No. Sampled	No. Positives	Infection prevalence (%)	Mean GE	GE Range
*Alsodes australis*	2	0	0	-	-
*Alsodes barrioi*	12	0	0	-	-
*Alsodes nodosus*	12	4	33.3	17.7	1.7−53.1
*Alsodes verrucosus*	2	0	0	-	-
*Batrachyla antartandica*	34	6	17.6	177.6	1.4−656.3
*Batrachyla leptopus*	16	0	0	-	-
*Batrachyla taeniata*	68	3	4.4	180.5	26.5−408.9
*Calyptocephalella gayi*	18	18	100	997.0	79.3−3,355.0
*Eupsophus altor*	1	0	0	-	-
*Eupsophus calcaratus*	32	1	3.1	5.4	-
*Eupsophus contulmoensis*	15	4	26.7	5.0	0.1−14.0
*Eupophus emiliopugini*	4	0	0	-	-
*Eupsophus nahuelbutensis*	59	3	5.1	15.1	0.4−40.1
*Eupsophus roseus*	10	2	20.0	6.4	2,6−11,7
*Eupsophus vertebralis*	1	0	0	-	-
*Hylorina sylvatica*	3	1	33.3	593.3	-
*Pleurodema thaul*	137	51	37.2	149.5	0.2−4,481.0
*Rhinoderma darwinii*	369	7	1.9	1,221.4	6.7−7,059.1
*Telmatobufo bullocki*	2	0	0	-	-

Genomic equivalents (GE) detected using a *Bd*-specific quantitative real-time PCR Taqman assay are expressed in means and ranges.

**Figure 2:**
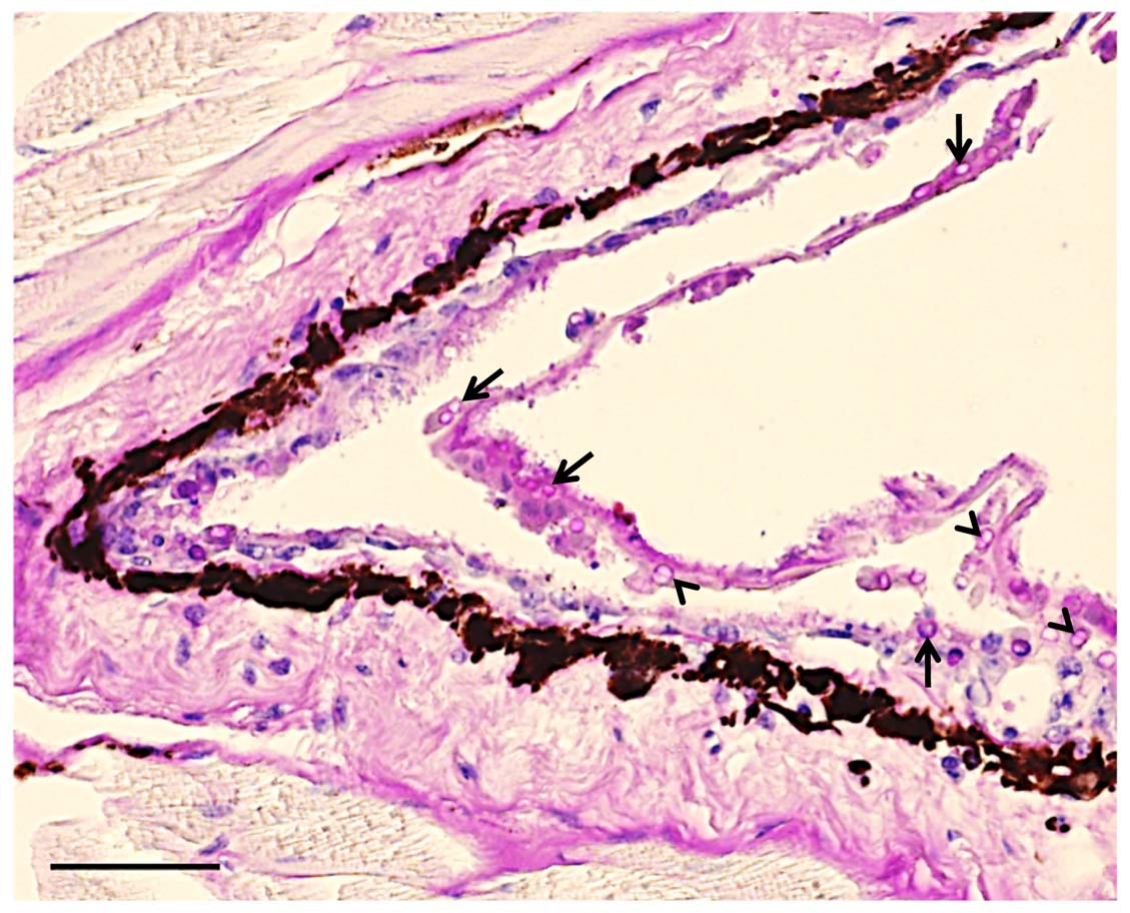
Skin histological section of a wild southern Darwin’s frog (*Rhinoderma darwinii*) with cutaneous chytridiomycosis. Note multiple empty zoosporangia (arrows) within the superficial keratinised layer of the epidermis. Several zoosporangia with an internal septum can be seen (arrowheads), morphologically typical of *Batrachochytrium dendrobatidis*. Stained with Periodic Acid-Shiff (PAS). Bar  =  20 µm.

Of the 369 *R. darwinii* tested, seven frogs from four different populations were positive for *Bd* (Table 4). Overall, the *Bd* prevalence in *R. darwinii* (1.9%) was significantly lower to that in sympatric amphibians tested (n = 109, 7.3%; *x* ^2^ = 8.200, *P* = 0.004). Contemporary *R. darwinii* populations sampled in south and southern Chile and their *Bd* prevalences are shown in Table 5. Although *R. darwinii* had the highest infection intensities (median: 127.1; range: 6.7−7,059.1 GE) when compared with all other infected species (13.9; 0.1−4,481.0 GE) they were not significantly different (Mann-Whitney *U*-test; U = 188.0, *P* = 0.063). Of particular interest were two *R. darwinii* from which GE counts over 1,000 were detected. Both frogs belonged to the northernmost known populations. Of these, one individual (NATRE74/12; 1,020 GE) was found dead at the capture site and subsequent histopathological examination revealed chytridiomycosis as the cause of death (Figure 2). The prevalence of *Bd* infection was significantly higher at sites with either *Rhinoderma* spp. extinction or severe population decline (30.0%) than at sites with no apparent *Rhinoderma* spp. declines (3.0%; *x* ^2^ = 106.407, *P*<0.001). Additionally, when *Bd* prevalence by site and geographical location were analysed, a linear regression revealed an inverse relationship between *Bd* prevalence and latitude (*R* ^2^ = 0.405, *P*<0.001; Figure 1).

**Table 4:** Details of *Batrachochytrium dendrobatidis* positive Southern Darwin’s frogs (*Rhinoderma darwinii*) sampled during the period 2008−2012 in south Chile with number of genomic equivalents (GE) detected using a *Bd*-specific quantitative real-time PCR Taqman assay.

Reference	Site	Animal	GE	SD^a^
NATRE74/12	El Natre	subadult	1,019.5	90.3
NATRE151/12	El Natre	brooding male	249.0	40.4
CON123/10	Contulmo	adult female	7,059.1	777.4
CON224/11	Contulmo	Juvenile	21.0	0.5
SD08/11	Senda Darwin	adult male	127.1	9.5
SD03/11	Senda Darwin	adult male	67.9	3.7
YAL45/12	Yaldad	adult female	6.7	0.3

a Standard deviation.

**Table 5:** *Batrachochytrium dendrobatidis* infection in 14 extant populations of the southern Darwin’s frog (*Rhinoderma darwinii*) sampled during the period 2009−2012 in south Chile.

Site	Coordinates (lat, long)	altitude (m)	No. sampled	No. positives	Infection prevalence (%)
Butamalal	37° 49' S, 73° 09' W	560	6	0	0
El Natre	37° 53' S, 73° 16' W	433	15	2	13,3
Contulmo	38° 01' S, 73° 11' W	370	13	2	15,4
PN Huerquehue	39° 08' S, 71° 42' W	1,239	1	0	0
PN Villarrica	39° 29' S, 71° 51' W	1,114	32	0	0
Coñaripe	39° 33' S, 71° 59' W	371	14	0	0
Oncol	39° 42' S, 73° 18' W	523	5	0	0
Huilo Huilo	39° 52' S, 71° 54' W	619	36	0	0
PN Alerce Costero	40° 12' S, 73° 26' W	912	7	0	0
PN Puyehue	40° 39' S, 72° 11' W	352	22	0	0
Senda Darwin	41° 53' S, 73° 41' W	9	14	2	14,3
Los Alerzales	42° 35' S, 74° 05' W	169	13	0	0
Tantauco Norte	43° 02' S, 73° 48' W	146	27	1	3,7
Tantauco Sur	43° 22' S, 74° 07' W	5	97	0	0
PN Queulat	44° 14' S, 72° 30' W	143	67	0	0

## Discussion

Museum amphibian specimens have been increasingly recognised as a valuable source of information for retrospective epidemiological studies [31], [32], [33], [34], [35]. Using such specimens, we demonstrated historical evidence of *Bd* infection in three species of native frogs from south Chile (*R. darwinii*, *R. rufum* and *P. thaul*). Although we examined similar numbers of frogs that had been collected prior to 1970 and post-1970, all six *Bd*-positive archived amphibians were collected from 1970 to 1978 inclusive: a time coincident with the onset of the global amphibian population decline phenomenon, including the disappearance of *R. rufum*, and the occurrence of the first amphibian global extinctions subsequently associated with *Bd* [16], [35], [36]. The only *R. rufum Bd*-positive animal was an individual kept in a jar with 179 other *R. rufum* specimens, all of which had been collected from Chiguayante (Biobío Region, near Concepción) during a two-day collection session in December 1975.

As the fixation history of the examined archived amphibians is not known, the overall infection prevalence (0.9%) and intensity of infection (GE values 0.1−0.6) obtained are likely an underestimation of the true situation. For example, although all of the archived specimens examined were preserved in alcohol, it is highly possible that many had been initially fixed in formalin, a chemical known to degrade DNA, reducing the likelihood of *Bd* detection [27]. Also, the fixative, IMS, can inhibit PCR. A previous study, however, was successful in detecting *Bd* DNA from the skin of amphibian specimens fixed in IMS [35] and in the current study we incorporated BSA to the PCR protocol to minimize the effect of any PCR inhibiters present [29].

Our field surveys failed to detect *R. rufum*, but infection with *Bd* was found in extant *R. darwinii*, but at a lower prevalence (1.9%) than in the other sympatric amphibian species tested (prevalence 7.3%), possibly as a consequence of different habitat use by the studied species (e.g. dependence of water for breeding). If highly susceptible to chytridiomycosis, however, it is possible that *R. darwinii* die soon after infection. This also would result in a low infection prevalence and might explain the disappearance of *Rhinoderma* spp. from many of the sites where *Bd* was found, especially if other amphibians act as reservoirs of infection, as might be predicted from their higher *Bd* prevalences [17], [37].

Amphibian chytridiomycosis is thought to have caused 100% mortality of 30 wild-caught *R. darwinii* exported to Germany in 2007 [23], [25]. According to these authors [25], travel stress and lack of isolation between individuals during transportation might have contributed to this high mortality rate. In the current study, two of seven *Bd*-positive wild *R. darwinii* had infection loads > 1,000 GE; including an individual found dead with chytridiomycosis. Disease and mortality caused by chytridiomycosis have been associated with infections higher than 1,000 GE in experimentally-infected green tree frogs (*Litoria caerulea*) [38], [39]. Experimental *Bd* infection trials in *R. darwinii*, similar to those performed with the Critically Endangered New Zealand Archey’s frog (*Leiopelma archeyi*) [40], [41] and with the Panamanian golden frog (*Atelopus zeteki*) [42], should be considered to further investigate the susceptibility of *R. darwinii* to chytridiomycosis. As the outcomes of *Bd* infection often are highly context-specific, experimental infection studies using *R. darwinii* under different hydric environments could help to infer the likely effects of *Bd* infection on *R. darwinii* under different climate and land-use change scenarios [43], [44], [45]. In a declining species like *R. darwinii*, however, promoting the survival of the species has to take priority: the use of animals in experiments should be internationally justifiable and only surplus captive-bred animals not suitable for conservation programmes should be used.

*Batrachochytrium dendrobatidis* is a waterborne pathogen and stream-living has been identified as a risk factor for *Bd*-associated declines [46]. *Rhinoderma darwinii* has evolved to develop an extreme case of parental care in which the species does not depend on water bodies for tadpole development [47]. In contrast, while *R. rufum* tadpoles spend their first two weeks of development in the vocal sacs of their male parents, they are then released into water as larvae where they live for the next approximately 120 days until metamorphosis takes place [48]. This association of *R. rufum* with streams in central Chile could render this species even more susceptible to population declines and extinction due to chytridiomycosis. Although found in only a single archived specimen, evidence of *Bd* infection was found in possibly the largest known *R. rufum* population [8] five years before the species was last recorded [5]. This, along with a positive association between *Bd* prevalence and *Rhinoderma* spp. population extinction/decline, suggests a possible association between chytridiomycosis and the disappearance of *R. rufum*.

We detected an inverse relationship between *Bd* prevalence and latitude, similar to that found by Kriger et al. [49] in the stony creek frog (*Litoria lesueuri*) in eastern Australia. Whether this is a reflection of the historical introduction and spread of *Bd* in Chile, with the organism not yet having reached the south of the country, or if it is due to environmental factors (e.g. temperature) is not yet clear. Longitudinal sampling of sites across the gradient would help to answer this question. That such a gradient exists, however, indicates that northern populations of *R. darwinii* are likely to be under a greater threat from chytridiomycosis than those in the south. It also suggests that the instigation of biosecurity measures might decrease the rate of spread of the disease to the southern populations of *R. darwinii* (assuming that *Bd* has not already reached this region and is less readily detected due to the low temperatures there limiting its growth).

It is not known if the *Bd* detected in the archived or extant specimens in the current study is the hypervirulent *Bd*GPL, a *Bd*GPL-hybrid, or perhaps an endemic lineage (or lineages) of the fungus. If *Bd*GPL is present in Chile, its spread to the country might have occurred via the introduction of *X. laevis* [32]. Feral populations of this invasive species, which have been established in central Chile since the 1970s, are known to be *Bd*-positive, although other mechanisms of pathogen introduction cannot be excluded [24].

## Conclusions

This is the first report of widespread *Bd* presence in Chile and our results provide evidence of an association between the presence of *Bd* and mortality in wild *R. darwinii*. Although, assessing the role of pathogens in extinctions remains problematic and infectious diseases are probably an underestimated cause of biodiversity loss [16], retrospective and prospective epidemiological data provide evidence that *Bd* infection is probably implicated in the enigmatic disappearance of *R. rufum* and the declines of *R. darwinii*, particularly from the northern part of their historical range. Nevertheless, further studies, such as the isolation and DNA sequencing of *Bd* in Chile, are required to further investigate the possible role of *Bd* in *Rhinoderma* spp. declines.
